# Supplementary material for: A morphotropic phase boundary in MA1−xFAxPbI3: linking structure, dynamics, and electronic properties
Source: Nat Commun. 2025 Oct 2;16:8775. doi: 10.1038/s41467-025-64526-4 (PMC12491417; doi:10.1038/s41467-025-64526-4)
Supplement: Supplementary file 1 — Supplementary Information [file 41467_2025_64526_MOESM1_ESM.pdf]

Supplementary Information

A Morphotropic Phase Boundary in $MA_{1-x}FA_xPbI_3$: Linking Structure, Dynamics, and Electronic Properties

Tobias Hainer, Erik Fransson, Sangita Dutta, Julia Wiktor, and Paul Erhart*
Department of Physics, Chalmers University of Technology, SE-41296, Gothenburg, Sweden
* erhart@chalmers.se

Contents

Supplementary Figures **1**
S1. Training of machine-learned interatomic potential . . . 1
S2. Evolution of weights during training . . . 2
S3. Distribution of weights after training . . . 2
S4. Parity plots for machine-learned interatomic potential . . . 3
S5. Direction of MA and FA bonds as a function of composition . . . 4
S6. Effective electron-phonon coupling strength compared to experiment . . . 4

Supplementary References **5**

Supplementary Figures

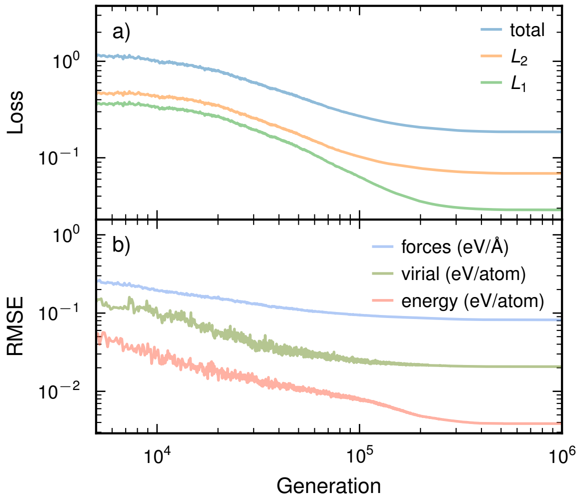

Fig. S1: Training of machine-learned interatomic potential. Evolution of (a) the total loss function $\mathcal{L}$ (blue) and the $\mathcal{L}_1$ (green) and $\mathcal{L}_2$ norms (orange) of the parameter vector as well as (b) the root mean square errors (RMSEs) of the energies $\mathcal{L}_\mathrm{e}$ (red), forces $\mathcal{L}_\mathrm{f}$ (blue), and virials $\mathcal{L}_\mathrm{v}$ (green). The total loss function is given by $\mathcal{L} = \lambda_1\mathcal{L}_1 + \lambda_2\mathcal{L}_2 + \lambda_e\mathcal{L}_\mathrm{e} + \lambda_f\mathcal{L}_\mathrm{f} + \lambda_v\mathcal{L}_\mathrm{v}$, where λ_i are hyperparameters of the optimization procedure.

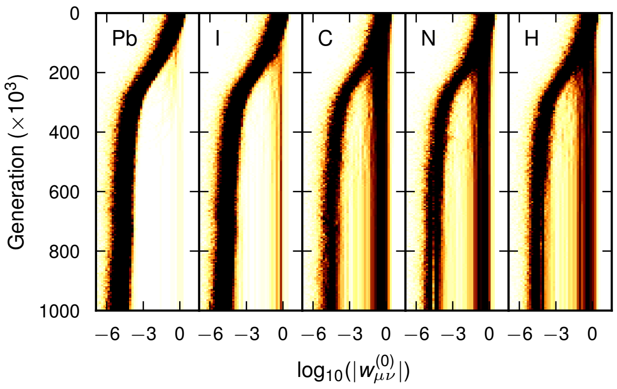

Fig. S2: Evolution of weights during training. Evolution of the absolute values of the weights $w_{\mu\nu}^{(0)}$ of the edges connecting the descriptor layer to the hidden layer of the neural network in the neuroevolution potential (NEP) model. The figure illustrates the effect of the $\mathcal{L}_1$ and $\mathcal{L}_2$ regularization terms in the NEP loss function (Figure S1), which enforce sparsity of the solution (Figure S3). By preventing accidental activation of features that were underrepresented during training during production runs this contributes to model stability and reliability.

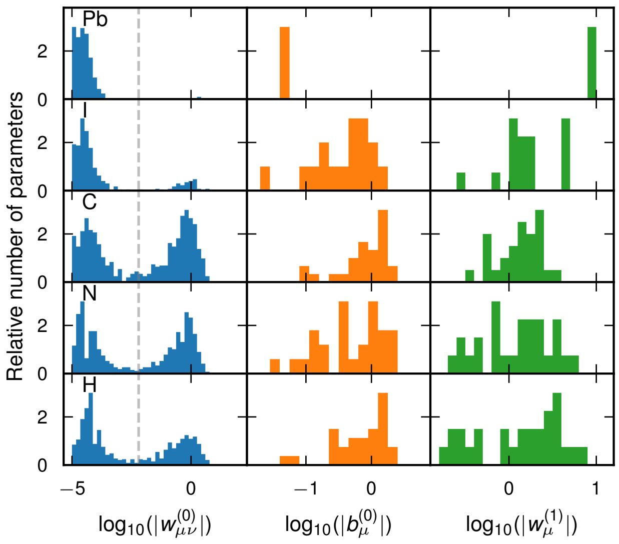

Fig. S3: Distribution of weights after training. Distribution of the absolute values of the neural network weights $w_{\mu\nu}^{(0)}$ (left), $b_{\mu}^{(0)}$ (center), and $w_{\mu}^{(1)}$ at the end of training. See Ref. 1 for the definition of these weights. The figure illustrates the effect of the $\mathcal{L}_1$ and $\mathcal{L}_2$ regularization terms in the NEP loss function (Figure S1), which enforce sparsity of the solution. The dashed vertical line in the left most column shows the separation between weights that are contributing (to the right of the vertical line) and that can be removed with negligible effect on the model scores (to the left of the vertical line). These weights can in principle be pruned and inactive edges and nodes can be pruned from the neural network, although this has not been done here.

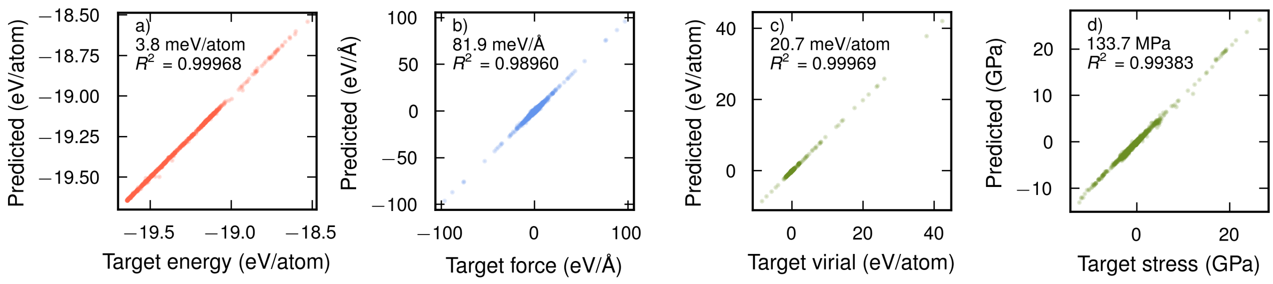

Fig. S4: Parity plots for machine-learned interatomic potential. Parity plots for (a) energy, (b) forces, (c) virials, and (d) stresses for the machine-learned interatomic potential of the NEP form constructed in this work. The insets indicate the RMSEs and coefficients of correlation (R^2).

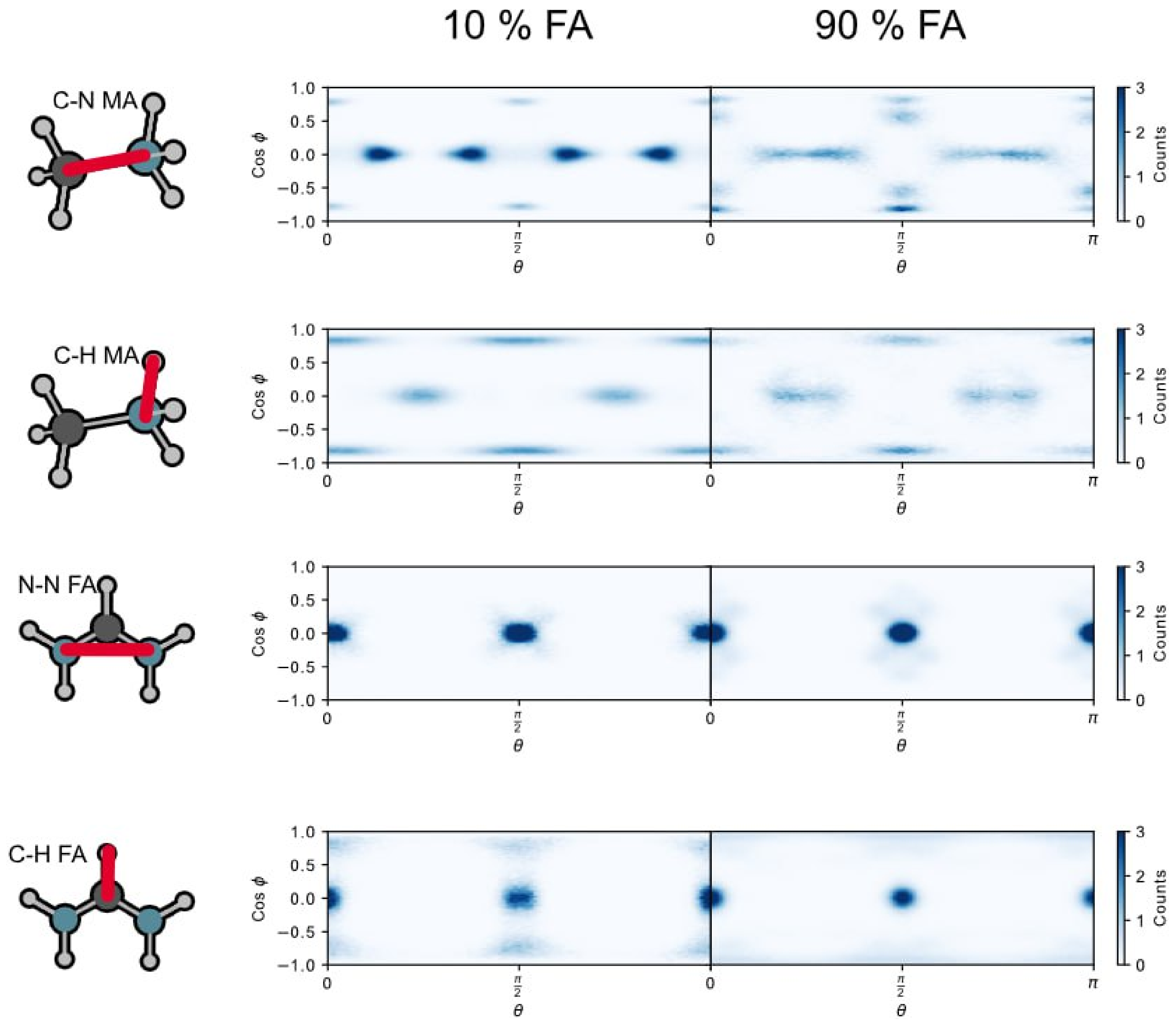

Fig. S5: Direction of MA and FA bonds as a function of composition. Bond distribution for C–N and C–H in MA, as well as N–N and C–H in FA at 10% and 90% FA fraction. While the distributions vary quantitatively with FA content there are no pronounced qualitative changes.

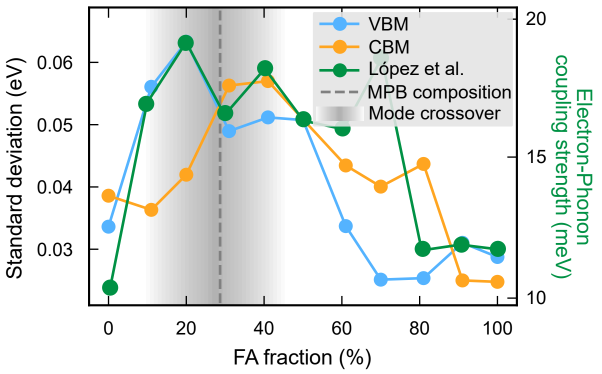

Fig. S6: Effective electron-phonon coupling strength compared to experiment. The experimental data are taken Ref. 2 There is a clear correlation between the fluctuations of the valence band maximum (VBM) and conduction band minimum (VBM) obtained from density functional theory (DFT) and the experimentally extracted electron-phonon coupling.

Supplementary References

[1] Z. Fan, Y. Wang, P. Ying, K. Song, J. Wang, Y. Wang, Z. Zeng, K. Xu, E. Lindgren, J. M. Rahm, A. J. Gabourie, J. Liu, H. Dong, J. Wu, Y. Chen, Z. Zhong, J. Sun, P. Erhart, Y. Su, and T. Ala-Nissila, *GPUMD: A package for constructing accurate machine-learned potentials and performing highly efficient atomistic simulations,* The Journal of Chemical Physics **157**, 114801 (2022). `doi:10.1063/5.0106617`.

[2] A. Francisco-López, B. Charles, M. I. Alonso, M. Garriga, M. Campoy-Quiles, M. T. Weller, and A. R. Goñi, *Phase Diagram of Methylammonium/Formamidinium Lead Iodide Perovskite Solid Solutions from Temperature-Dependent Photoluminescence and Raman Spectroscopies,* The Journal of Physical Chemistry C **124**, 3448 (2020). `doi:10.1021/acs.jpcc.9b10185`.
